# Deep Learning-Based Alzheimer’s Disease Detection from Multi-Channel EEG Using Fused Time–Frequency Image Grids

**DOI:** 10.3390/diagnostics16050746

**Published:** 2026-03-02

**Authors:** Abdulnasır Yıldız, Hasan Zan

**Affiliations:** 1 Department of Electrical and Electronics Engineering, Dicle University, Diyarbakır 21200, Turkey; 2 Department of Computer Engineering, Mardin Artuklu University, Mardin 47200, Turkey; hasanzan@artuklu.edu.tr

**Keywords:** dementia detection, electroencephalography (EEG), time–frequency representation, deep learning, multi-channel image fusion

## Abstract

**Background/Objectives:** Dementia is a progressive neurodegenerative disorder for which accurate and timely diagnosis remains a major clinical challenge. Electroencephalography (EEG) offers a noninvasive and cost-effective means of capturing neurophysiological alterations, motivating the development of reliable EEG-based automated diagnostic frameworks. This study aims to systematically examine how different time–frequency representations (TFRs) affect dementia classification performance within a unified multi-channel EEG image fusion framework. **Methods:** Resting-state, eyes-closed EEG recordings from 88 subjects, including Alzheimer’s disease, frontotemporal dementia, and cognitively normal controls, were preprocessed and segmented. Channel-wise signals were converted into two-dimensional time–frequency images using Short-Time Fourier Transform (STFT), Continuous Wavelet Transform (CWT), Hilbert–Huang Transform (HHT), Wigner–Ville Distribution (WVD), or Constant-Q Transform (CQT). Images from 19 EEG channels were fused into a structured grid and classified using pretrained convolutional neural networks, including MobileNetV2, ResNet-50, and InceptionV3. **Results:** Results indicate that classification performance is highly dependent on the chosen TFR. The STFT-based representation combined with InceptionV3 achieved the highest accuracy, reaching 98.8% with random splitting and 84.3% with subject-wise splitting, outperforming previous studies. CQT also showed competitive performance, whereas HHT and WVD were less effective. Gradient-weighted class activation mapping provided interpretable visualization of physiologically relevant EEG channel contributions. **Conclusions:** The proposed framework demonstrates the importance of structured multi-channel fusion and systematic TFR evaluation for robust and interpretable EEG-based dementia classification and serves as a foundation for future cross-dataset validation.

## 1. Introduction

Dementia encompasses a group of progressive neurodegenerative conditions that impair memory, cognition, and functional independence [1], and it has become one of the most pressing global public health challenges of the twenty-first century [2]. With global aging, dementia prevalence is rising sharply, imposing unsustainable medical, socioeconomic, and caregiver burdens worldwide [3]. Among the various dementia subtypes, Alzheimer’s disease (AD) is the most widespread, characterized by gradual declines in memory, executive function, and learning ability due to widespread neurodegeneration [4,5]. Frontotemporal dementia (FTD), though less prevalent than AD, predominantly strikes individuals under 65, causing severe behavioral disinhibition, language deficits, and dysexecutive syndrome—distinct profiles that often delay or confuse diagnosis [6,7]. Both disorders lack curative treatments, making early, reliable, and accessible detection essential for timely clinical intervention, patient management, caregiver planning, and the evaluation of emerging therapeutic strategies [8].

Despite advances in clinical practice, current diagnostic pathways for dementia rely heavily on neuropsychological evaluations, structured cognitive assessments, and neuroimaging techniques such as magnetic resonance imaging (MRI), computed tomography (CT), and positron emission tomography (PET) [9,10]. Although these tools provide valuable insights into structural and functional brain alterations, they are often time-consuming, expensive, and dependent on specialized expertise, which limits their availability in many clinical settings [11,12]. Neuroimaging modalities may also involve exposure to radiation or require the administration of radioactive tracers, adding further constraints for routine or repeated use [13]. As a result, substantial diagnostic delays are common, particularly in resource-limited environments, reducing the effectiveness of early intervention strategies [14]. These challenges have intensified efforts to identify alternative biomarkers that are non-invasive, cost-effective, widely accessible, and capable of capturing disease-related neural changes at early stages of cognitive decline [15,16].

Electroencephalography (EEG) has emerged as a particularly promising modality for dementia assessment due to its non-invasive nature, relatively low cost, and exceptional temporal resolution, which enables the capture of rapid neural dynamics that are not easily observable through other techniques [17,18]. Numerous studies have reported characteristic EEG alterations in individuals with AD and FTD, including slowed dominant rhythms, reduced complexity, altered synchronization, and disrupted connectivity patterns across cortical regions [19,20,21]. These electrophysiological signatures suggest that EEG can offer valuable biomarkers for distinguishing between dementia subtypes and healthy cognitive function. Conventional EEG analysis, typically based on visual inspection or handcrafted spectral and statistical features, can reveal such disease-related patterns; however, these approaches are often limited by subjectivity, inter-rater variability, and reduced sensitivity to subtle, high-dimensional temporal–spectral interactions. As a result, there has been a growing shift toward computational approaches capable of modeling complex EEG dynamics more effectively [22].

Building on this computational shift, studies leveraging EEG for dementia assessment generally fall into two broad methodological categories: traditional machine learning pipelines that rely on handcrafted feature extraction, and deep learning frameworks designed to learn discriminative representations directly from the data. Within the machine learning paradigm, researchers typically construct structured workflows in which informative descriptors of EEG activity are manually engineered before being used to train classifiers such as support vector machines [23,24], k-nearest neighbors [23,25], decision trees [26], ensemble methods [27,28], or linear discriminant analysis [29]. These handcrafted features often aim to capture spectral power variations, temporal complexity, nonlinear dynamics, or entropy-based characteristics of the signal [23,30]. A substantial body of work has demonstrated the diagnostic potential of such approaches, with many studies reporting accuracies in the 86–96% range. Although machine learning-based strategies established the feasibility of EEG-driven dementia classification, their dependence on manual feature engineering and their susceptibility to dataset-specific tuning revealed inherent limitations that ultimately motivated the adoption of deep learning approaches capable of automated feature learning.

On the other hand, deep learning has become widely recognized for its ability to extract intricate and clinically relevant patterns from diverse biomedical datasets, including EEG [31,32]. In recent years, there has been an increasing emphasis on leveraging deep learning methods to analyze EEG recordings for neurological disorders such as AD and FTD because of their automated feature-extraction capability and improved representation learning [33,34]. Deep learning models can capture complex temporal and spectral dependencies directly from raw or minimally transformed EEG signals, reducing or eliminating the need for extensive handcrafted feature engineering. Several studies have demonstrated this potential. Ferri et al. [35] employed stacked autoencoders to learn latent representations from resting-state EEG, achieving 80% accuracy in distinguishing AD from healthy controls (HC). Alessandrini et al. [36] trained recurrent neural networks (RNNs) to model temporal dependencies in EEG and reported 79.3% accuracy on a cohort of 35 subjects. Miltiadous et al. [37] further introduced DICE-Net, a dual-input architecture combining convolutional, transformer encoder, and feed-forward layers to classify AD and FTD using band-power and coherence features, achieving 83.28% accuracy for AD vs. HC and 74.96% for FTD vs. HC. Chen et al. [38] combined convolutional neural network (CNN) and vision transformer (ViT) branches to improve representation learning, reaching 87.33% accuracy for AD vs. HC and 82.98% for FTD vs. HC on the same dataset. These studies collectively highlight the promise of deep learning frameworks operating on raw or transformed EEG signals.

Complementing these advances, a separate line of research has focused on transforming EEG signals into rich two-dimensional time–frequency representations (TFRs) prior to deep learning analysis. These approaches rely on the premise that TFRs provide a more informative depiction of the non-stationary characteristics of EEG, enabling convolutional models to better capture localized spectral–temporal patterns. Fouladi et al. [39] converted EEG recordings into RGB images using continuous wavelet transform and trained a custom CNN on data from 180 subjects, achieving an accuracy of 92%. Ieracitano et al. [40] employed power spectral density (PSD) maps as grayscale images and used CNN to classify 126 subjects (63 AD, 63 HC), achieving 93% accuracy. More recently, Stefanou et al. [41] used Fourier transform-based spectrograms with CNN architecture and demonstrated improved cross-subject generalization, obtaining accuracies of 79.5% for AD versus controls and 80.7% for AD + FTD versus controls. Collectively, these studies underscore the value of TFRs in enhancing discriminative learning in EEG-based dementia detection. However, existing work typically relies on a single TFR, limits the use of spatially diverse EEG channels, or lacks a systematic framework for comparing alternative representations. Consequently, it remains unclear which TFRs offer the most informative features for distinguishing AD, FTD, and healthy cognition, highlighting a critical gap that motivates the present study.

Despite these promising developments, important gaps remain in the existing literature. Current studies typically rely on a single TFR or focus on limited channel subsets, leaving open the question of how different TFRs compare when evaluated within a unified and controlled framework. Moreover, only a few deep learning approaches explicitly integrate information from all EEG channels in a structured manner that preserves the spatial relationships across the scalp, which is essential for capturing distributed pathological patterns characteristic of AD and FTD. As a result, it is still not well understood which TFRs provide the most discriminative and neurologically meaningful features for distinguishing between AD, FTD, and HC. Addressing these shortcomings requires a systematic, multi-representation analysis capable of jointly leveraging the full spatial richness of multi-channel EEG.

In light of these gaps, the present study aims to develop a unified and comprehensive framework for dementia detection using EEG by proposing a novel multi-channel image fusion pipeline and systematically evaluating multiple TFRs within a controlled deep learning setting. Specifically, we examine five widely used TFRs—Short-Time Fourier Transform (STFT), Continuous Wavelet Transform (CWT), Hilbert–Huang Transform (HHT), Wigner–Ville Distribution (WVD), and Constant-Q Transform (CQT)—and benchmark their performance using three state-of-the-art pretrained CNNs: ResNet-50, InceptionV3, and MobileNetV2. This design enables a direct, fair, and reproducible comparison of how different TFRs contribute to the classification of AD and FTD.

## 2. Material and Methods

This section describes the complete computational pipeline proposed for EEG-based dementia detection. It details the dataset and preprocessing procedures, the generation of channel-wise TFRs, the construction of the fused multi-channel image input, and the deep learning architectures and training strategies used for model development and evaluation.

### 2.1. Dataset

The present study uses a publicly available EEG dataset [42], and all clinical diagnoses and cognitive assessments were performed during the original data collection. Alzheimer’s disease and frontotemporal dementia diagnoses were established by the dataset authors according to standard clinical criteria, including Diagnostic and Statistical Manual of Mental Disorders, 3rd ed., revised (DSM-IIIR, DSM IV, ICD-10) and the National Institute of Neurological, Communicative Disorders and Stroke—Alzheimer’s Disease and Related Disorders Association (NINCDS—ADRDA) guidelines. The Mini-Mental State Examination (MMSE) was administered as part of the original clinical evaluation to quantify overall cognitive status and disease severity across groups. No diagnostic procedures or cognitive testing were conducted by the authors of the present study, and no additional neuropsychological measures are available within the dataset.

**Figure 1:**
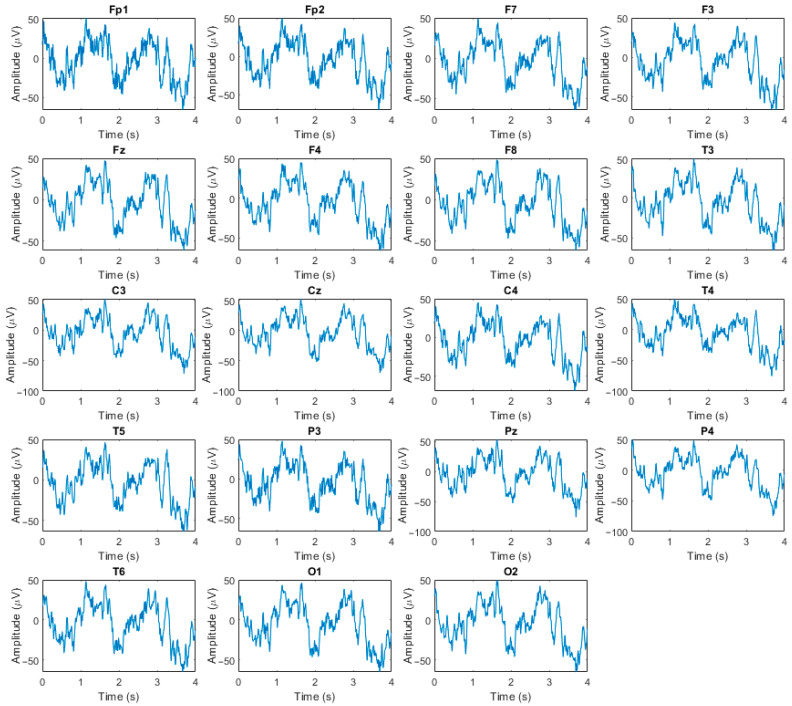
Representative example of EEG recordings acquired from a single subject.

All EEG recordings were obtained during routine clinical examinations conducted in a hospital environment using a Nihon Kohden 2100 EEG system (Nihon Kohden Corporation, Tokyo, Japan). Signals were acquired from 19 scalp electrodes positioned according to the international 10–20 system (Fp1, Fp2, F7, F3, Fz, F4, F8, T3, C3, Cz, C4, T4, T5, P3, Pz, P4, T6, O1, and O2), with mastoid electrodes A1 and A2 used for impedance monitoring. The original dataset documentation does not report a specific quantitative impedance threshold; impedance checks were performed as part of the routine clinical acquisition protocol. Data were recorded in a referential montage using Cz as the online reference, while participants were seated comfortably and instructed to keep their eyes closed. The EEG was sampled at 500 Hz with a resolution of 10 µV/mm. To ensure signal quality, subsequent preprocessing procedures, including band-pass filtering, Artifact Subspace Reconstruction, and Independent Component Analysis, were applied to mitigate potential artifacts and variability associated with acquisition conditions. Representative EEG signals from a single subject are illustrated in Figure 1.

The dataset comprises three diagnostic groups: AD, FTD, and HC. Clinical diagnoses for AD and FTD were established by experienced clinicians according to internationally accepted criteria, including Diagnostic and Statistical Manual of Mental Disorders and the National Institute of Neurological, Communicative Disorders and Stroke—Alzheimer’s Disease and Related Disorders Association guidelines. Cognitive status was assessed using the Mini-Mental State Examination (MMSE), a 30-point standardized screening tool in which scores of 24–30 are generally considered indicative of normal cognition, scores between 18–23 correspond to mild cognitive impairment, and scores below 18 are associated with moderate to severe cognitive impairment [43]. Accordingly, lower MMSE scores reflect greater levels of global cognitive dysfunction. The HC group exhibited normal cognitive function, with MMSE scores of 30.

The AD group consists of 36 participants (12 males and 24 females) with a mean age of 66.4 ± 7.9 years and an average MMSE score of 17.75, reflecting moderate cognitive decline. The FTD cohort includes 23 individuals (14 males and 9 females) with a mean age of 63.6 ± 8.2 years and a mean MMSE score of 22.17. The HC group comprises 29 neurologically healthy subjects (11 males and 18 females) with a mean age of 67.9 ± 5.4 years, all achieving the maximum MMSE score. Recording durations varied across subjects but were comparable across diagnostic groups. AD recordings had a mean duration of approximately 13.5 ± 2.70, FTD recordings averaged 12.0 ± 1.87, and HC recordings were slightly longer, with a mean duration of 13.8 ± 2.14. In total, the dataset contains 485.5 min of AD EEG, 276.5 min of FTD EEG, and 402 min of HC EEG data.

A detailed summary of demographic and clinical characteristics, including age ranges, sex distribution, MMSE statistics, and group-wise statistical comparisons, is provided in Table 1.

In line with previous studies [37,41,44], the EEG recordings were first band-pass filtered using a Butterworth filter with cutoff frequencies of 0.5 and 45 Hz to suppress slow drifts and high-frequency artifacts. Following filtering, the signals were offline re-referenced to the average of the O1 and O2 electrodes, replacing the original online Cz reference used during acquisition. Artifact Subspace Reconstruction was then applied to attenuate transient or high-amplitude artifacts using a conservative sliding window of 0.5 s and a maximum allowed standard deviation threshold of 17. Subsequently, Independent Component Analysis was performed to decompose the EEG into statistically independent sources, and components automatically identified as ocular or muscle-related artifacts based on their spatial topographies and temporal characteristics were removed, consistent with established ICA-based artifact rejection approaches [45,46]. The recordings were acquired under eyes-closed resting-state conditions, which are commonly used in routine clinical EEG examinations to reduce visual stimulation and task-related variability and are frequently adopted in EEG-based dementia research [47,48]. Although recordings were obtained in this controlled condition, residual eye movement and blink-related activity was still present in several sessions and was effectively suppressed through this artifact removal pipeline.

After artifact removal, the cleaned EEG signals were resampled from 500 Hz to 125 Hz. This step substantially reduces computational and memory demands during time–frequency transformation and deep learning training, while preserving the frequency content relevant to dementia-related EEG analysis, which predominantly lies below 45 Hz. Following resampling, and in line with [49], the signals were segmented into overlapping 20-s windows with 50% overlap.

Ethical approval was not applicable for this study, as no new data was collected by the authors. The EEG data used are fully anonymized and publicly available, and were obtained from an existing dataset released for research purposes.

### 2.2. Overview

**Figure 2:**
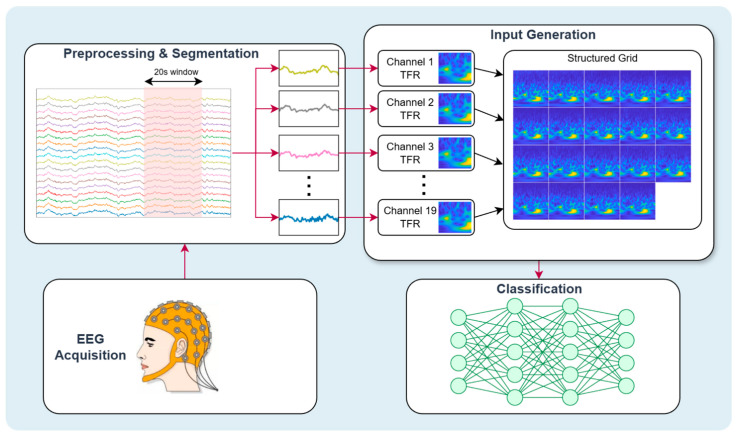
Overview of the proposed EEG–image fusion framework for dementia detection. The pipeline includes EEG preprocessing and segmentation, channel-wise time–frequency transformation using alternative TFR methods, fusion of the resulting images into a structured multichannel representation, and classification using pretrained deep learning models.

This study proposes a complete EEG–image fusion pipeline designed to transform multichannel EEG recordings into informative TFRs suitable for deep learning-based dementia detection. As illustrated in Figure 2, the framework begins with EEG acquisition followed by preprocessing and segmentation, as described in Section 2.1. Each segmented window from the 19 channels is then independently transformed into one of five candidate TFRs—STFT, CWT, HHT, WVD, or CQT—depending on the experimental configuration. Every channel-level TFR is rendered as a standardized RGB image, and these images are arranged into a structured grid to form a single fused representation of the multichannel segment. The resulting composite image is subsequently fed into pretrained CNNs, including MobileNet-v2, ResNet-50, and Inception-v3, which leverage transfer learning to extract discriminative spectral–spatial features and perform dementia classification.

### 2.3. Input Generation

**Figure 3:**
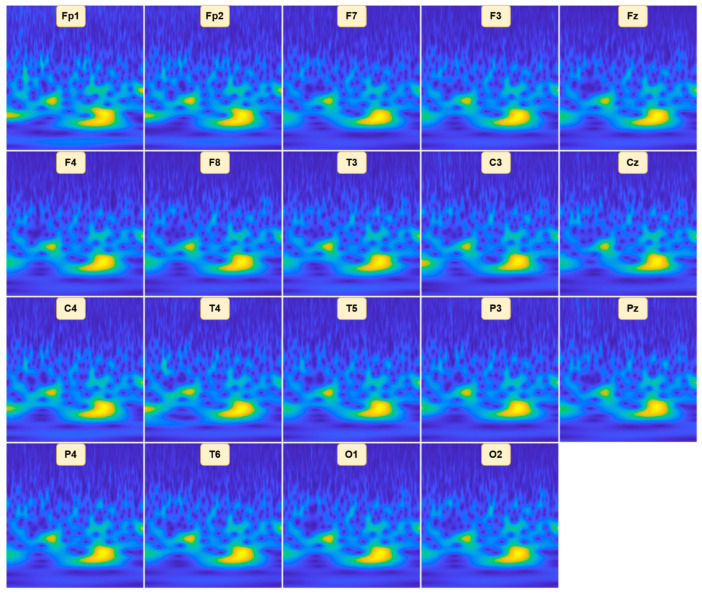
Example of a fused EEG time–frequency representation constructed by arranging channel-wise TFR images into a fixed 4 × 5 grid.

After preprocessing and segmentation, each of the 19 EEG channels is converted into a time–frequency representation using one of the five methods considered in this study. To construct a unified input suitable for CNNs, all channel-wise TFR images are placed into a fixed 4 × 5 grid, following a predefined sequence that preserves channel identity and maintains consistency across samples. As illustrated in Figure 3, the first row contains the frontal electrodes Fp1, Fp2, F7, F3, and Fz; the second row includes F4, F8, T3, C3, and Cz; the third row arranges C4, T4, T5, P3, and Pz; and the final row includes P4, T6, O1, O2, with the remaining grid cell left empty. Each channel’s TFR is rendered as an RGB image using uniform resolution and color scaling. The resulting fused image integrates spectral–temporal information from all channels into a single composite representation that can be directly processed by the deep learning models employed in this study.

#### 2.3.1. Short-Time Fourier Transform

Short-Time Fourier Transform (STFT) is a technique commonly used for analyzing nonstationary signals by representing their frequency content over successive short time intervals [50]. By applying the Fourier transform within sliding windows, STFT enables localized time–frequency analysis, which is well suited for capturing the dynamic spectral behavior of EEG signals. In discrete form, STFT of a signal x[n] is defined as:

(1) $$STFT\left(m,k\right)=\sum_{n=0}^{N-1}x\left[n\right]w\left[n-m\right]e^{-j2\pi kn/N}$$

where w[⋅] denotes the window function centered at time index m, k is the frequency bin index, and N is the window length. The magnitude of the STFT coefficients provides a time–frequency representation describing the distribution of signal energy across time and frequency.

In this work, STFT was computed using a Hanning window with a duration of 0.5 s and 50% overlap between consecutive windows. This parameter configuration was chosen to balance temporal localization and frequency resolution, allowing meaningful characterization of EEG rhythms while maintaining continuity between adjacent time frames. Such a compromise is particularly important for dementia-related EEG analysis, where both transient and sustained oscillatory patterns are informative.

#### 2.3.2. Continuous Wavelet Transform

Continuous Wavelet Transform (CWT) provides a multiresolution time–frequency analysis by decomposing a signal into scaled and shifted versions of a prototype waveform, known as the mother wavelet [51]. Unlike fixed-resolution methods such as STFT, CWT offers adaptive time and frequency resolution, making it well suited for capturing both slow and fast oscillatory components of nonstationary EEG signals. For a discrete-time signal x[n], CWT is expressed as:

(2) $$CWT\left(a,b\right)=\sum_{n=0}^{N-1}x\left[n\right]\frac{1}{\sqrt{a}}\;\psi^{*}\left(\frac{n-b}{a}\right)$$

where ψ(⋅) denotes the mother wavelet, a is the scale parameter controlling frequency resolution, b represents the time shift, ${\left(\cdot \right)}^{*}$ denotes complex conjugation, and N is the signal length. Smaller scales correspond to higher frequencies with finer temporal localization, while larger scales capture lower-frequency components with improved frequency resolution.

In this study, the analytic Morse (amor) wavelet was selected due to its strong time–frequency localization properties and analytic nature, which reduce spectral leakage and enable accurate frequency estimation. The scale discretization was configured with ten voices per octave, providing a dense frequency sampling that facilitates the capture of subtle spectral–temporal variations in EEG activity.

#### 2.3.3. Hilbert–Huang Transform

Hilbert–Huang Transform (HHT) is a data-driven time–frequency analysis technique specifically designed for nonlinear and nonstationary signals [52]. Unlike transform-based methods that rely on predefined basis functions, HHT adaptively decomposes the signal according to its intrinsic oscillatory modes.

HHT consists of two main stages. First, Empirical Mode Decomposition (EMD) is applied to decompose a discrete EEG signal x[n] into a finite set of intrinsic mode functions (IMFs) and a residual component:

(3) $$x\left[n\right]=\sum_{k=1}^{K}IMF_{k}\left[n\right]+r\left[n\right]$$

where ${IMF}_{k}[n]$ denotes the k-th intrinsic mode function, r[n] is the residual, and K is the total number of extracted IMFs. Each IMF represents a narrowband oscillatory component with locally symmetric envelopes and a well-defined instantaneous frequency. In this study, the maximum number of intrinsic mode functions was limited to 40 based on preliminary experiments assessing decomposition stability across EEG segments. This upper bound was found to provide sufficient decomposition depth to capture relevant oscillatory components while avoiding over-fragmentation, mode mixing, and unstable instantaneous frequency estimates that can arise when excessively large numbers of IMFs are extracted.

To improve the numerical stability of the envelope estimation during EMD, piecewise cubic Hermite interpolation was employed when constructing the upper and lower envelopes from local extrema. This interpolation strategy preserves local monotonicity and reduces overshooting artifacts, resulting in smoother and more physically meaningful IMFs.

In the second stage, the Hilbert transform is applied to each IMF to compute the corresponding analytic signal:

(4) $$z_{k}\left[n\right]={IMF}_{k}\left[n\right]+j\;H\left\{{IMF}_{k}\left[n\right]\right\}$$

where H{⋅} denotes the Hilbert transform and j is the imaginary unit. From the analytic signal, the instantaneous amplitude and instantaneous frequency are derived, enabling the construction of the Hilbert spectrum, which represents the signal’s energy distribution over time and frequency.

#### 2.3.4. Wigner-Ville Distribution

Wigner–Ville Distribution (WVD) is a quadratic time–frequency representation that provides high-resolution characterization of signal energy jointly in time and frequency [53]. For a discrete-time EEG signal x[n], the classical WVD is defined as:

(5) $$W_{x}\left[n,f\right]=\sum_{m=-M}^{M}x\left[n+m\right]x^{*}\left[n-m\right]e^{-j2\pi fm}$$

where n denotes the time index, f represents frequency, ${\left(\cdot \right)}^{*}$ denotes complex conjugation, and m is the lag variable. WVD is known for its excellent localization properties; however, when applied to multicomponent signals such as EEG, it may produce cross-term interference that can obscure meaningful spectral structures.

To mitigate this effect, a smoothed pseudo-Wigner–Ville formulation was adopted in this study. In this variant, smoothing windows are applied along both the time and lag dimensions prior to frequency transformation, effectively suppressing cross-terms while preserving the dominant auto-term components associated with genuine signal oscillations. This trade-off yields a cleaner and more interpretable time–frequency representation, which is particularly important for nonstationary EEG data.

The time–frequency plane was discretized using 2500 uniformly spaced time samples and 1000 frequency bins. These values were selected based on empirical evaluations to ensure adequate temporal and spectral resolution for capturing EEG dynamics within each segment, while maintaining numerical stability and manageable computational cost. Increasing the resolution beyond these values did not yield perceptible improvements in representation quality, whereas lower resolutions resulted in loss of fine spectral–temporal detail. The adopted configuration therefore represents a practical balance between resolution, stability, and efficiency.

#### 2.3.5. Constant-Q Transform

Constant-Q Transform (CQT) is a time–frequency representation designed to provide logarithmically spaced frequency bins, offering higher frequency resolution at lower frequencies and finer temporal resolution at higher frequencies [54]. This property makes CQT particularly suitable for analyzing EEG signals, whose diagnostically relevant information is often concentrated in lower frequency bands. Unlike linear-frequency transforms, CQT maintains a constant quality factor Q, defined as the ratio of center frequency to bandwidth. For a discrete-time EEG signal x[n], CQT coefficient at time index n and frequency bin k can be expressed as

(6) $$X\left(k,n\right)=\sum_{m=0}^{L_{k}-1}x\backslash \left[n-m\right]w_{k}{\left[m\right]e}^{-j2\pi \frac{f_{k}}{f_{s}}m\;}$$

where $f_{s}$ denotes the sampling frequency, $f_{k}$ is the center frequency of the k-th frequency bin, $w_{k}[m]$ is a frequency-dependent analysis window, and $L_{k}$ is the window length associated with that bin. The window length varies inversely with frequency, ensuring that the ratio $Q=\frac{f_{k}}{\Delta f_{k}}$ remains constant across all bins.

In this study, the frequency axis was partitioned into 24 bins per octave, resulting in fine-grained logarithmic frequency resolution. This choice enables detailed modeling of EEG oscillatory activity across conventional frequency bands, such as delta, theta, alpha, beta, and gamma, while preserving sensitivity to subtle frequency shifts that may be associated with dementia-related neurophysiological changes.

For all five time–frequency representation methods considered in this study, the transformations were applied independently to each EEG channel within the segmented signals described at the beginning of Section 2.3. The resulting time–frequency coefficients were converted into two-dimensional images using a consistent resolution and normalization strategy to ensure comparability across representations and channels. For STFT, CWT, and CQT, the magnitude (amplitude) of the time–frequency coefficients was used to generate the corresponding images, whereas for HHT and WVD, the resulting time–frequency maps represent energy distributions derived from instantaneous amplitude or quadratic energy formulations.

Linear magnitude scaling was applied to STFT-, HHT-, and WVD-based representations, while logarithmic frequency scaling is inherently employed in CWT and CQT due to their multiresolution and constant-Q formulations. For all representations, the resulting amplitude or energy values were normalized to the range of $\left[0,\;1\right]$ prior to visualization and mapped to a uniform RGB colormap, ensuring consistent intensity encoding across channels and time–frequency methods. These channel-wise RGB images were then combined according to the predefined grid arrangement to form a single fused multichannel representation, which serves as the final input to the deep learning models. This unified image generation and fusion process enables a fair and systematic evaluation of alternative TFRs within an identical learning framework.

### 2.4. Transfer Learning and Models Employed

Transfer learning has become a widely adopted strategy in deep learning applications where labeled data are limited, as is often the case in biomedical signal analysis [55]. Instead of training deep neural networks from scratch, transfer learning leverages knowledge acquired from large-scale datasets to initialize model parameters, allowing the network to adapt more effectively to a target task. In this study, transfer learning is particularly advantageous because the proposed framework converts multichannel EEG recordings into standard three-channel RGB images, enabling the direct use of well-established two-dimensional CNNs originally developed for natural image recognition.

All TFRs generated through the proposed pipeline were mapped to RGB image format and resized to match the input requirements of the selected pretrained architectures. This design choice allows the models to exploit rich hierarchical feature extractors learned from large image repositories, while fine-tuning the higher-level layers to capture EEG-specific temporal–spectral patterns associated with dementia. Compared to EEG-specific or one-dimensional architectures, this approach benefits from mature, well-validated network designs and stable optimization behavior.

Three pretrained CNNs were employed in this study: MobileNetV2 [56], ResNet-50 [57], and InceptionV3 [58]. These models represent different points along the accuracy–efficiency spectrum and enable a systematic comparison between lightweight and more computationally intensive architectures. MobileNetV2 is designed for efficiency and employs depthwise separable convolutions and inverted residual blocks, substantially reducing the number of trainable parameters and floating-point operations while maintaining competitive representational capacity. ResNet-50 introduces deep residual learning through skip connections, allowing effective training of deeper networks and improved feature discrimination at the cost of increased computational complexity. InceptionV3 utilizes parallel convolutional kernels of varying sizes within its inception modules, enabling multi-scale feature extraction but requiring higher computational resources.

Table 2 reports the number of trainable parameters and the approximate number of floating-point operations (FLOPs) required for a single forward pass for each architecture under the adopted input resolution. This comparison allows an explicit assessment of the trade-off between representational capacity and computational efficiency among the evaluated models.

### 2.5. Model Fine-Tuning

All pretrained networks were fine-tuned following a unified training protocol to ensure a fair and consistent comparison across models and TFRs. The segments were randomly partitioned into training, validation, and test sets using a 60%–20%–20% split, a ratio that has been commonly adopted in prior EEG-based deep learning studies to balance model learning, hyperparameter tuning, and unbiased performance evaluation [59]. The training set was used to update network weights, the validation set to monitor generalization behavior and guide early stopping, and the test set was reserved exclusively for final performance assessment.

Fine-tuning was performed using the Adam optimizer with an initial learning rate set to $5\times 10^{-4}$, which provided stable convergence while allowing sufficient adaptation of pretrained weights to the EEG-based image domain. The networks were trained for a maximum of 200 epochs using the cross-entropy loss function, appropriate for multi-class dementia classification. To mitigate overfitting and improve robustness, online data augmentation was applied during training using only structure-preserving transformations. Specifically, small random translations along the time and frequency axes and mild isotropic scaling were employed to simulate natural temporal misalignments and global amplitude variations commonly observed in EEG recordings. In addition, reflection was applied along the time axis only, corresponding to a time-reversal operation that does not alter spectral content or inter-channel relationships. No transformations that would invert or distort the frequency axis were used, ensuring that the temporal–spectral interpretation of the TFRs is preserved.

An early stopping mechanism was employed based on validation accuracy, with training terminated if no improvement was observed for 20 consecutive epochs. This strategy prevents unnecessary training and reduces the risk of overfitting, particularly given the limited size of clinical EEG datasets. All experiments were implemented and conducted using MATLAB R2024b, ensuring a reproducible and standardized experimental environment across all model configurations.

### 2.6. Model Evaluation

The performance of the proposed framework was assessed using standard classification metrics that are widely adopted in medical decision-support systems. Specifically, accuracy, macro-averaged precision, macro-averaged recall, and macro-averaged F1-score were employed to provide a comprehensive evaluation of model behavior across all classes. Macro-averaging was chosen to ensure that each class contributes equally to the final score, which is particularly important in dementia classification scenarios where class imbalance may exist. Performance metrics and confusion matrices reported in this study were computed at the segment (window) level, reflecting the direct predictions produced by the models from individual TFRs.

Accuracy measures the overall proportion of correctly classified samples and is defined as

(7) $$Accuracy\left(ACC\right)=\frac{TP+TN}{TP+TN+FP+FN}$$

where TP, TN, FP, and FN represent the numbers of true positives, true negatives, false positives, and false negatives, respectively.

Precision quantifies the reliability of positive predictions by measuring the proportion of correctly identified samples among all samples predicted for a given class. Recall reflects the model’s ability to correctly identify all samples belonging to a particular class. For multi-class evaluation, precision and recall were computed independently for each class and then averaged using macro-averaging, which assigns equal weight to all classes regardless of their sample sizes. Macro-averaged precision and recall are defined as

(8) $$Precision\left(PR\right)=\frac{1}{C}\sum_{i=1}^{C}\frac{TP_{i}}{TP_{i}+FP_{i}}$$

(9) $$Recall\left(RE\right)=\frac{1}{C}\sum_{i=1}^{C}\frac{TP_{i}}{TP_{i}+FN_{i}}$$

where C denotes the number of classes and $TP_{i}$, $FP_{i}$, and $FN_{i}$ correspond to class-specific true positives, false positives, and false negatives.

The macro-averaged F1-score provides a balanced measure that jointly considers precision and recall and is computed as the harmonic mean of the macro-averaged precision and recall:

(10) $$F1-score\left(F1\right)=\frac{2\times PR\times RE}{PR+RE}$$

## 3. Results

This section presents the experimental results obtained from the proposed multi-channel EEG image fusion framework for dementia detection. Classification performance is evaluated across all combinations of TFRs and pretrained deep learning architectures using the held-out test set described in Section 2.5. Standard evaluation metrics, including accuracy, macro-averaged precision, recall, and F1-score, are reported to enable a comprehensive and balanced assessment of model behavior across classes.

The results obtained using STFT are summarized in Table 3. STFT-based representations yielded consistently strong performance across all three pretrained models, indicating that the localized spectral–temporal information captured by this representation is highly discriminative for dementia classification. All models achieved accuracy values exceeding 97%, with closely aligned macro-averaged precision, recall, and F1-score, reflecting balanced classification behavior across the target classes.

Among the evaluated architectures, Inception achieved the highest overall performance when trained on STFT-based inputs, attaining an accuracy of 98.76%. ResNet-50 and MobileNet also demonstrated competitive performance, with accuracies of 97.88% and 97.67%, respectively. The relatively small performance gap between models suggests that, when paired with an effective time–frequency representation such as STFT, the proposed input generation and fusion strategy enables robust feature learning even with lightweight architectures.

Table 4 presents the classification performance of MobileNetV2, ResNet-50, and InceptionV3 when using CWT-based EEG image representations on the test set. All models demonstrate strong performance, with InceptionV3 achieving the highest accuracy of 97.52%. ResNet-50 and MobileNetV2 show closely aligned performance, with accuracies of 96.86% and 95.84%, respectively. Precision, recall, and F1-scores follow a similar trend, with all models performing consistently across these metrics. These results highlight the effectiveness of CWT-based representations for dementia classification.

Table 5 summarizes the classification results obtained using HHT-based EEG image representations on the test set. Among the evaluated models, InceptionV3 achieves the highest performance, with an accuracy of 92.49%, followed by MobileNetV2 at 91.76%. ResNet-50 attains a comparatively lower accuracy of 89.42%, yet still maintains stable precision, recall, and F1-score values. The consistent alignment across all evaluation metrics indicates reliable class-wise predictions for each model when HHT-derived representations are employed.

Table 6 reports the classification performance achieved using WVD-based EEG image representations on the test set. Overall, the results indicate a noticeable reduction in classification accuracy compared to the previously reported representations, reflecting the reduced discriminative power of WVD-derived features. Among the evaluated architectures, InceptionV3 yields the highest accuracy of 84.32%, followed by ResNet-50 with 80.89%, while MobileNetV2 attains an accuracy of 78.56%. For all models, precision, recall, and F1-score remain identical, indicating consistent predictive behavior across classes when WVD-based inputs are used.

Table 7 summarizes the classification results obtained using CQT-based EEG image representations on the test set. The results demonstrate strong and stable performance across all three pretrained architectures, indicating that the constant-Q representation effectively captures discriminative spectral–temporal characteristics of EEG signals. InceptionV3 achieves the highest performance with an accuracy of 97.52%, followed by MobileNetV2 at 96.35% and ResNet-50 at 95.19%. As observed for the other representations, precision, recall, and F1-score are equal for each model, suggesting balanced classification behavior across the evaluated classes when using CQT-based inputs.

**Figure 4:**
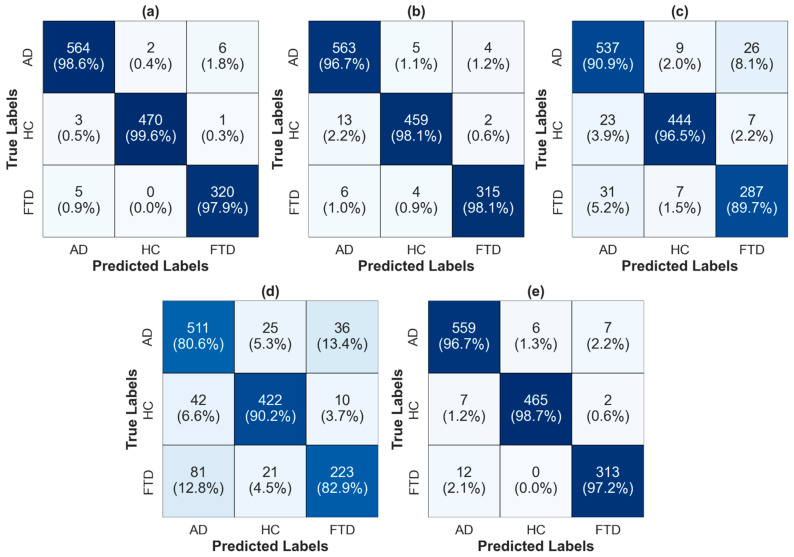
Confusion matrices obtained using the InceptionV3 model, which achieved the highest accuracy for each time–frequency representation: (**a**) STFT, (**b**) CWT, (**c**) HHT, (**d**) WVD, and (**e**) CQT. Each cell shows the number of samples, with percentages in parentheses indicating the proportion relative to the total samples of the corresponding true class. Color intensity reflects classification performance, where darker diagonal cells indicate higher correct classification rates and lighter off-diagonal cells denote misclassification among AD, HC, and FTD.

Figure 4 presents the confusion matrices obtained using the InceptionV3 architecture, which consistently achieved the highest classification accuracy for each time–frequency representation and is therefore reported as the best-performing model in all cases. The matrices provide a detailed class-wise evaluation for AD, HC, and FTD. Each cell reports the absolute number of test samples, while the percentages in parentheses denote the proportion of correctly or incorrectly classified samples relative to the total number of instances in the corresponding true class, i.e., row-wise percentages. Darker color shades along the main diagonal indicate higher true positive rates, whereas lighter off-diagonal cells reflect lower misclassification frequencies.

In STFT-based setting (Figure 4a), the model achieves very high correct classification rates across all classes, with AD, HC, and FTD recognition exceeding 97%, and only a small number of misclassifications primarily occurring between AD and FTD. A similarly strong pattern is observed for CWT-based representation (Figure 4b), where diagonal dominance remains pronounced and class confusion is minimal, indicating robust separability among the three groups. In contrast, HHT-based results (Figure 4c) show a noticeable increase in confusion, particularly between AD and FTD, reflected by reduced diagonal percentages for both classes. WVD-based confusion matrix (Figure 4d) exhibits the highest level of misclassification, with substantial overlap between AD and FTD and a comparatively lower correct classification rate for all classes, consistent with its overall reduced accuracy. Finally, the CQT-based representation (Figure 4e) restores strong class discrimination, with high true positive rates for AD, HC, and FTD and minimal cross-class errors. Overall, the confusion matrices corroborate the quantitative performance metrics by highlighting how different TFRs influence class separability, with STFT, CWT, and CQT yielding the most reliable and balanced predictions across dementia categories.

## 4. Discussion

This section discusses the experimental findings obtained from the proposed multi-channel EEG image fusion framework. By systematically evaluating five different TFRs under a unified deep learning setup, the results allow a direct assessment of how spectral–temporal encoding choices influence classification performance, independent of preprocessing, data splits, and training strategy.

**Figure 5:**
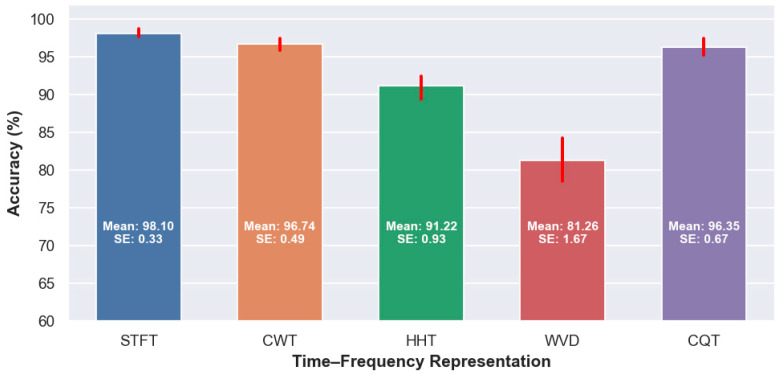
Mean classification accuracy (%) achieved by different TFRs (STFT, CWT, HHT, WVD, and CQT) across all pretrained deep learning models on the test set. Each bar represents the mean accuracy for a given representation, while the overlaid red markers indicate the variability of performance across models, highlighting the consistency and robustness of each time–frequency representation. All values are expressed in percentages. SE denotes the standard error of the mean.

As shown in Figure 5, noticeable performance differences emerge across the evaluated TFRs, underscoring the critical role of spectral–temporal encoding in EEG-based dementia detection. STFT consistently yields the highest accuracy, which can be attributed to its stable and compact spectral representation that effectively captures dementia-related EEG phenomena such as dominant rhythm slowing and band-specific power redistribution. While CWT also achieves strong performance, its results are slightly inferior to STFT. This marginal decrease can be explained by the presence of redundant and overlapping components inherent to multiscale wavelet decompositions, where neighboring scales may encode highly correlated information. Such redundancy can dilute discriminative patterns and reduce learning efficiency, particularly when channel-wise representations are fused into a single composite image.

In contrast, WVD exhibits the weakest performance among the evaluated TFRs, as observed by its lower accuracy distribution in Figure 5. Although WVD offers high theoretical time–frequency resolution, its practical application to multicomponent EEG signals is challenging. In particular, the smoothing window commonly employed to suppress cross-term interference effectively acts as a low-pass filter, which may inadvertently attenuate or remove diagnostically relevant high-frequency components [60]. As a result, even smoothed WVD representations can yield distorted or incomplete spectral patterns, limiting the ability of deep networks to extract discriminative features. HHT demonstrates moderate performance but remains less competitive, which aligns with its well-documented sensitivity to noise, mode mixing, and signal variability [61]. The adaptive nature of empirical mode decomposition can result in inconsistent intrinsic mode functions across subjects, reducing the robustness of the derived time–frequency patterns.

Notably, CQT achieves performance comparable to STFT and outperforms both HHT and WVD, as reflected in Figure 5. This strong performance can be attributed to CQT’s logarithmic frequency scaling, which provides higher frequency resolution at lower frequencies and finer temporal resolution at higher frequencies, closely matching the spectral organization of EEG rhythms. Importantly, CQT achieves this without introducing redundant content across scales, resulting in compact and discriminative representations well suited for deep learning. Overall, the cross-TFR analysis in Figure 5 highlights that representations offering a balanced trade-off between resolution, stability, and non-redundancy are most effective for EEG-based dementia classification.

**Figure 6:**
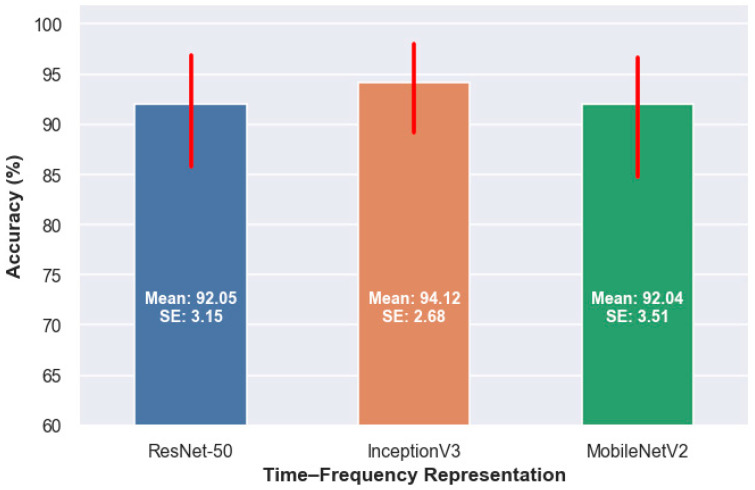
Mean classification accuracy (%) of ResNet-50, InceptionV3, and MobileNetV2 aggregated across all TFRs. Bars indicate the mean accuracy for each model, while the overlaid red markers reflect the distribution of performance across different representations, illustrating both central tendency and variability in model behavior. All values are expressed in percentages. SE denotes the standard error of the mean.

As shown in Figure 6, InceptionV3 achieves the highest median accuracy across all TFRs, followed by MobileNetV2 and ResNet-50. InceptionV3 contains approximately 23.9 million trainable parameters, and its multi-scale inception modules enable effective modeling of complex and heterogeneous EEG time–frequency patterns, which explains its superior performance.

MobileNetV2, with about 3.4 million parameters, attains a median accuracy slightly lower than InceptionV3 but higher than ResNet-50. Despite its lightweight design, the use of depthwise separable convolutions and inverted residual blocks allows it to preserve discriminative features efficiently, making it an attractive choice when computational efficiency is a priority. ResNet-50, with roughly 25.6 million parameters, shows the lowest median accuracy among the three models in this study. Although its residual connections facilitate stable training of deep networks, its higher complexity does not translate into superior performance for the considered EEG image representations.

### 4.1. Robustness Analysis

To assess the robustness and generalizability of the proposed framework, additional experiments were conducted using the best-performing configuration identified in earlier Section 3, namely the InceptionV3 classifier combined with STFT-based TFRs. The evaluation was repeated using 10-fold cross-validation under two different data-splitting strategies: a conventional random split at the segment level and a subject-wise split designed to prevent information leakage across subjects.

**Figure 7:**
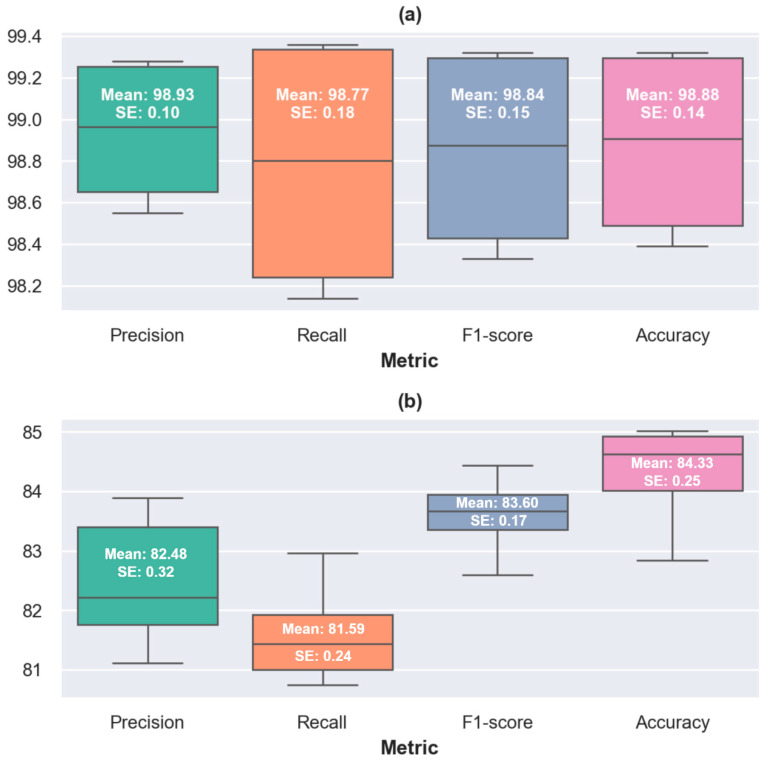
Box plots summarizing the robustness analysis of the best-performing configuration (InceptionV3 with STFT-based TRRs) under two evaluation protocols: (**a**) random segment-level data splitting and (**b**) subject-wise data splitting. Each box plot illustrates the distribution of classification performance across 10-fold cross-validation in terms of accuracy, precision, recall, and F1-score, highlighting the differences in performance stability and inter-fold variability between the two splitting strategies. All values are expressed in percentages. SE denotes the standard error of the mean.

Under the random split protocol, EEG segments were randomly partitioned into training and test sets while maintaining class balance. The model achieved a mean accuracy of 98.88% and a mean macro-averaged F1-score of 98.85% across the 10 folds. The 90% confidence intervals were ±0.25% for accuracy and ±0.27% for the F1-score, indicating highly consistent performance. The distribution of fold-wise results is illustrated in Figure 7a, where the narrow interquartile range reflects limited variability across folds.

For the subject-wise split, EEG segments were grouped by subject, and all segments belonging to a given subject were assigned exclusively to either the training set or the test set. This evaluation yielded a mean accuracy of 84.34% and a mean macro-averaged F1-score of 83.60%, with 90% confidence intervals of ±0.45% and ±0.32%, respectively. As shown in Figure 7b, a wider dispersion is observed compared to the random split, reflecting increased inter-subject variability in EEG characteristics.

The observed reduction in performance relative to the random split is expected and can be attributed to the strong subject-specific nature of EEG signals, including inter-individual differences in baseline rhythms, spectral power distribution, and noise characteristics. Under subject-wise evaluation, the model is required to generalize to entirely unseen subjects rather than to unseen segments from familiar subjects, which constitutes a substantially more challenging classification scenario. Nevertheless, the relatively narrow confidence intervals indicate stable performance across folds, suggesting that the proposed structured multi-channel fusion framework captures disease-related spectral–temporal patterns that generalize beyond subject-specific features.

Although model predictions were generated at the segment level, subject-level decisions can be obtained by aggregating the predictions of all segments associated with the same subject. In practice, this can be implemented using majority voting or by averaging posterior class probabilities across segments, resulting in a single diagnostic outcome per subject.

Overall, these results demonstrate that the proposed structured multi-channel fusion framework maintains stable performance across repeated evaluations and exhibits reliable generalization when tested on previously unseen subjects. The reported confidence intervals further support the statistical stability of the observed performance trends.

### 4.2. Grad-CAM Visualizations

Gradient-weighted Class Activation Mapping (Grad-CAM) is a post hoc interpretability technique that highlights the spatial regions of an input image that contribute most strongly to a model’s prediction. By computing the gradients of the target class score with respect to the feature maps of the final convolutional layer, Grad-CAM produces a coarse localization map indicating class-discriminative regions. When overlaid on the original input, warmer colors (e.g., red and yellow) correspond to regions with higher importance for the decision, whereas cooler colors (e.g., blue) indicate lower contribution. In this study, Grad-CAM is employed to provide insight into how the best-performing model, InceptionV3, utilizes the fused EEG time–frequency images for dementia classification.

**Figure 8:**
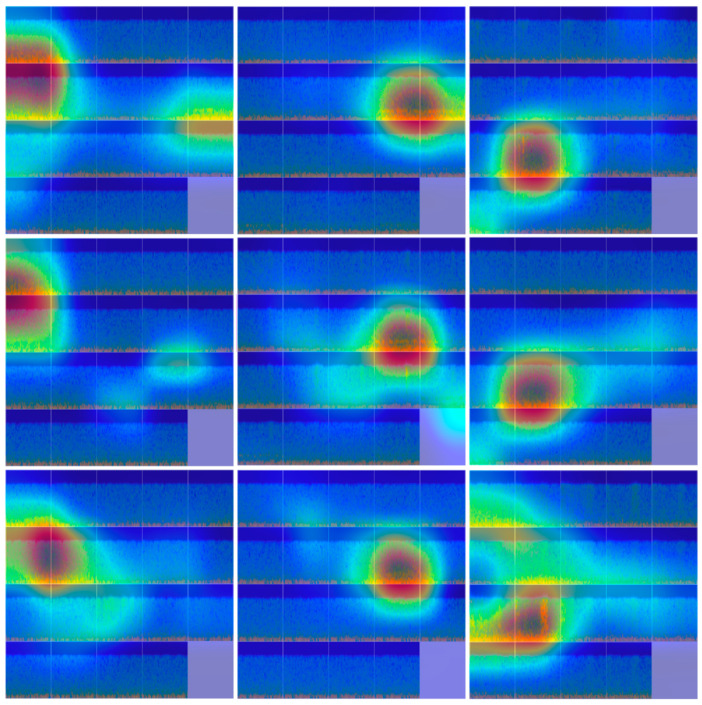
Grad-CAM visualizations generated using STFT-based EEG image representations and the InceptionV3 model. Each column corresponds to a diagnostic group, with AD shown in the first column, HC in the second column, and FTD in the third column. Warmer color shades (yellow–red) indicate regions that contribute more strongly to the model’s decision, whereas cooler shades (blue) denote lower relevance. The activation patterns highlight class-specific channel importance, with AD mainly emphasizing frontal electrodes (Fp1, F4, F8), HC showing stronger responses over central–parietal regions (C3, Cz, P3, Pz), and FTD exhibiting pronounced activations in temporal areas (T4, T5, T6).

As illustrated in Figure 8, distinct and class-dependent activation patterns are observed across the three diagnostic groups. For AD, Grad-CAM maps consistently emphasize frontal electrode regions, particularly Fp1, F4, and F8. This finding aligns with well-documented frontal dysfunction and reduced executive control associated with AD [62], suggesting that the model effectively captures disease-relevant frontal EEG alterations.

In contrast, HC subjects exhibit dominant activations over central and parietal regions, most notably C3, Cz, P3, and Pz. These areas are commonly associated with preserved sensorimotor and posterior cognitive processing [63,64], indicating that the model relies on stable and distributed spectral patterns characteristic of healthy brain activity.

For FTD, the strongest activations are localized over temporal electrodes, including T4, T5, and T6. This spatial emphasis is neurophysiologically consistent with the hallmark temporal lobe degeneration observed in FTD [65], demonstrating that the model leverages region-specific EEG signatures relevant to the underlying pathology.

### 4.3. Comparison with Previous Studies

A substantial body of work has investigated EEG-based dementia classification using the same clinically collected dataset employed in this study, enabling a meaningful comparison of methodological choices rather than data-related effects. As summarized in Table 8, prior studies differ not only in feature representation and model design, but also—critically—in their data-splitting strategies, which has a significant impact on reported performance.

Several earlier studies rely on random segment-level data splitting, where EEG segments are randomly assigned to training and test sets without enforcing subject exclusivity. Under this evaluation protocol, both classical machine learning and deep learning approaches have reported high classification accuracies. For example, Senkaya et al. [44] achieved 96.0% accuracy using handcrafted time-domain and PSD features with an SVM classifier. Similarly, CNN-based models operating on spectrogram or frequency-domain representations have reported accuracies above 87% under random splits. While these results demonstrate the discriminative potential of EEG-derived features, random splitting may benefit from subject-specific signal characteristics shared between training and test sets.

In contrast, fewer studies adopt subject-wise splitting, in which all segments from a given subject are assigned exclusively to either the training or test set. This protocol provides a more stringent and clinically relevant evaluation of generalization to unseen subjects. Among the studies reporting subject-wise results on this dataset, CNN–Transformer-based frameworks using band power or connectivity representations have achieved accuracies up to 80.2% for multi-class dementia classification. These results reflect the increased difficulty of subject-level generalization in EEG analysis.

Within this context, the proposed framework demonstrates competitive performance under both evaluation paradigms. As shown in Table 8, the proposed method achieves 98.8% accuracy under random segment-level splitting, exceeding previously reported results using the same protocol. More importantly, under subject-wise splitting, the proposed approach attains an accuracy of 84.3%, outperforming existing subject-wise studies on this dataset. The consistent performance gain across both splitting strategies highlights the effectiveness of the structured multi-channel time–frequency fusion scheme.

Unlike many prior deep learning studies that focus on a single representation or employ limited channel integration, the present work systematically evaluates multiple TFRs within a unified framework while explicitly accounting for differences in data-splitting strategies. This controlled evaluation clarifies the relationship between reported performance and validation protocol, and demonstrates that structured multi-channel fusion can improve generalization beyond subject-specific EEG patterns.

### 4.4. Limitations

Despite the encouraging results, several limitations of the present study should be acknowledged. First, the proposed 4 × 5 fixed grid fusion strategy, while effective for integrating all 19 EEG channels into a unified image representation, does not explicitly model the true spatial topology or anatomical neighborhood relationships among electrodes on the scalp. As a result, spatial dependencies are implicitly learned by the convolutional networks rather than being explicitly encoded, and alternative graph-based or topology-aware fusion schemes may further enhance spatial interpretability and performance.

Second, all experiments were conducted on a single publicly available dataset acquired using one EEG system, a fixed electrode configuration, and a uniform acquisition protocol under eyes-closed resting-state conditions. The overall sample size is also modest, which may restrict statistical power and generalizability. Although robustness analyses based on cross-validation and confidence intervals were performed to mitigate overfitting and dataset-specific bias, the reported results may not fully generalize to data collected with different hardware, montages, sampling rates, recording durations, or task paradigms such as eyes-open or task-based EEG. Consequently, the proposed framework should be regarded as a proof of concept that demonstrates the feasibility and potential benefits of systematic time–frequency representation comparison and structured multi-channel fusion, rather than as a definitive clinical solution.

Finally, the study primarily focuses on classification performance and representation-level comparisons rather than establishing direct neurophysiological correlations between EEG characteristics and clinical severity measures. While Grad-CAM visualizations provide qualitative insights into channel relevance, more detailed neurophysiological interpretation and longitudinal validation remain beyond the scope of the current work.

Future research will address these limitations by extending the framework to multi-center and cross-dataset evaluations, incorporating data recorded under diverse experimental conditions, and exploring topology-aware fusion mechanisms and subject-level modeling strategies. Such extensions are essential steps toward improving generalizability and facilitating eventual clinical translation of EEG-based dementia assessment systems.

## 5. Conclusions

This study presents a unified deep learning framework for EEG-based dementia detection through a systematic comparison of alternative TFRs within a structured multi-channel image fusion scheme. The results indicate that classification performance is influenced by the choice of representation, with STFT, CWT, and CQT providing more discriminative features than WVD and HHT under the evaluated setting, while InceptionV3 achieved the strongest overall performance among the pretrained models. Grad-CAM visualizations offered additional insight into channel-level relevance, supporting the neurophysiological plausibility of the learned representations. While the reported results demonstrate the potential of the proposed framework, further validation on larger, multi-center, and cross-dataset EEG cohorts is required to establish robustness, generalizability, and clinical applicability.

## Figures and Tables

**Table 1:** Demographic and clinical characteristics of the study population. The table reports age (minimum, maximum, and mean), sex distribution, and MMSE scores (minimum, maximum, and mean) for the AD, FTD, and HC groups. Group-wise statistical comparisons were performed using one-way ANOVA, and corresponding *p*-values are provided.

Characteristic		AD	FTD	HC	*p*-Value
**Sample size (*n*)**		36	23	29	-
**Age (years)**	Min.	49	44	57	0.115
	Max.	79	78	78	
	Mean ± SD	66.4 ± 7.9	63.6 ± 8.2	67.9 ± 5.4	
**Sex**	Female (*n*)	24	9	11	-
	Male (*n*)	12	14	18	
**MMSE score**	Min.	4	18	30	<0.001
	Max.	23	27	30	
	Mean ± SD	17.75 ± 4.50	22.2 ± 8.22	30.0 ± 0.0	
**Duration (min)**	Min.	5.1	7.9	12.5	0.556
	Max.	21.3	16.9	16.5	
	Mean ± SD	13.5 ± 2.70	12.0 ± 1.87	13.8 ± 2.14	

**Table 2:** Computational complexity of the employed models.

Model	Trainable Parameters (M)	FLOPs (G)
MobileNetV2	3.4	0.3
ResNet-50	25.6	4.1
InceptionV3	23.8	5.7

**Table 3:** Classification performance of MobileNetV2, ResNet-50, and InceptionV3 using STFT-based EEG image representations on the test set.

Model	Precision	Recall	F1-Score	Accuracy
MobileNet	97.61	97.54	97.57	97.67
ResNet-50	97.75	97.79	97.77	97.88
Inception	98.68	98.74	98.71	98.76

**Table 4:** Classification performance of MobileNetV2, ResNet-50, and InceptionV3 using CWT-based EEG image representations on the test set.

Model	Precision	Recall	F1-Score	Accuracy
MobileNet	95.95	95.64	95.79	95.84
ResNet-50	96.99	96.50	96.73	96.86
Inception	97.65	97.40	97.52	97.52

**Table 5:** Classification performance of MobileNetV2, ResNet-50, and InceptionV3 using HHT-based EEG image representations on the test set.

Model	Precision	Recall	F1-Score	Accuracy
MobileNet	91.70	91.16	91.40	91.76
ResNet-50	89.13	89.15	89.14	89.42
Inception	92.36	91.95	92.14	92.49

**Table 6:** Classification performance of MobileNetV2, ResNet-50, and InceptionV3 using WVD-based EEG image representations on the test set.

Model	Precision	Recall	F1-Score	Accuracy
MobileNet	78.04	76.80	77.30	78.56
ResNet-50	79.66	79.84	79.73	80.89
Inception	84.56	82.33	83.14	84.32

**Table 7:** Classification performance of MobileNetV2, ResNet-50, and InceptionV3 using CQT-based EEG image representations on the test set.

Model	Precision	Recall	F1-Score	Accuracy
MobileNet	96.28	96.02	96.15	96.35
ResNet-50	95.10	94.80	94.94	95.19
Inception	97.55	97.38	97.46	97.52

**Table 8:** Comparison of EEG-based dementia classification studies using the same dataset.

Study	EEG Representation	Learning Paradigm	Model	Data Split	Classification Task	Accuracy
Miltiadous et al. [37]	Band powers and coherence	DL	CNN–Transformer	Subject-wise	AD vs. HC FTD vs. HC	83.3 75.0
Chen et al. [38]	Raw EEG and frequency spectrum	DL	CNN–Transformer	Subject-wise	AD vs. FTD vs. HC	80.2
Stefanou et al. [41]	2D spectrogram images	DL	CNN	Subject-wise	AD vs. HC AD + FTD vs. HC	87.8 93.5
Senkaya et al. [44]	Time domain and PSD features	ML	SVM, k-NN	Random	AD vs. FTD vs. HC	96.0
Velichko et al. [66]	Raw EEG and discreate wavelet transform	ML	Neural Network Entropy	Random	AD vs. HC	88.5
Ma et al. [67]	Connectivity features	ML	SVM	Subject-wise	AD vs. HC FTD vs. HC AD vs. FTD	76.9 90.4 96.6
Rostamikia et al. [68]	Time domain, frequency domain, and connectivity features	ML	SVM	Random	AD vs. FTD AD + FTD vs. HC	87.8 93.5
Kang et al. [69]	3D spectro-spatial representation	DL	3D CNN	Subject-wise	AD vs. HC AD vs. HC AD + FTD vs. HC	74.2 77.1 73.3
**This study**	STFT, CWT, HHT, WVD, CQT	DL	CNN	Random	AD vs. FTD vs. HC	98.8
**This study**	STFT, CWT, HHT, WVD, CQT	DL	CNN	Subject-wise	AD vs. FTD vs. HC	84.3

PSD: Power Spectral Density, SVM: Support Vector Machines, k-NN: k-nearest Neighbors, ML: machine learning, DL: deep learning.

## Data Availability

The original data presented in the study are openly available at https://openneuro.org/datasets/ds006036/versions/1.0.5 (accessed on 10 September 2025).
